# On the Deployment and Noise Filtering of Vehicular Radar Application for Detection Enhancement in Roads and Tunnels

**DOI:** 10.3390/s18030837

**Published:** 2018-03-11

**Authors:** Young-Duk Kim, Guk-Jin Son, Chan-Ho Song, Hee-Kang Kim

**Affiliations:** Center for Future Automotive Convergence Research, DGIST, 333 Techno Jungang-daero, Hyeonpung-myeon, Dalseong-gun, Daegu 711-873, Korea; sudopop@dgist.ac.kr (G.-J.S.); realhyuk@dgist.ac.kr (C.-H.S.); heekangkim@dgist.ac.kr (H.-K.K.)

**Keywords:** radar sensor, radar installation, roadside and tunnel, noise filtering

## Abstract

Recently, radar technology has attracted attention for the realization of an intelligent transportation system (ITS) to monitor, track, and manage vehicle traffic on the roads as well as adaptive cruise control (ACC) and automatic emergency braking (AEB) for driving assistance of vehicles. However, when radar is installed on roads or in tunnels, the detection performance is significantly dependent on the deployment conditions and environment around the radar. In particular, in the case of tunnels, the detection accuracy for a moving vehicle drops sharply owing to the diffuse reflection of radio frequency (RF) signals. In this paper, we propose an optimal deployment condition based on height and tilt angle as well as a noise-filtering scheme for RF signals so that the performance of vehicle detection can be robust against external conditions on roads and in tunnels. To this end, first, we gather and analyze the misrecognition patterns of the radar by tracking a number of randomly selected vehicles on real roads. In order to overcome the limitations, we implement a novel road watch module (RWM) that is easily integrated into a conventional radar system such as Delphi ESR. The proposed system is able to perform real-time distributed data processing of the target vehicles by providing independent queues for each object of information that is incoming from the radar RF. Based on experiments with real roads and tunnels, the proposed scheme shows better performance than the conventional method with respect to the detection accuracy and delay time. The implemented system also provides a user-friendly interface to monitor and manage all traffic on roads and in tunnels. This will accelerate the popularization of future ITS services.

## 1. Introduction

Recently, as the traffic environment and transportation have been improved, the number of roads and tunnels interconnecting cities has increased accordingly. However, such an increase in the use of transportation infrastructure also causes traffic and safety management problems such as traffic jams and vehicle accidents. To exploit these issues, Intelligent Transportation System (ITS) technologies [1] have become very popular for monitoring traffic situations with high accuracy and in real-time. The most representative ITS technologies for sensing vehicular traffic are cameras, laser scanners and radar. Among these sensors, in general the radar sensor guarantees a robust performance for vehicle localization especially in bad weather conditions (e.g., rain, snow, smoke, clouds). Although the Global Positioning System (GPS), which uses a signal from satellites, can provide accurate localization and speed measurements, it does not work in tunnels or underground environments. In addition, radar systems are less affected by illumination, while camera and laser sensors suffer from performance degradations due to low illumination (e.g., at night). With the help of radar technology, in the past, many radar based sensor systems have been proposed for detecting and monitoring vehicle movements in road environments [2,3,4,5,6,7]. For example, Tan-Jan et al. [3] proposed a microwave radar detector which reduces noise signal and side lobes within each traffic lane by using Fast Fourier Transforms (FFTs) and time stamp information. Yichao et al. [4] proposed another vehicle detection scheme with a Gaussian mixture model for mitigating the interference in complex urban scenes such as trees and buildings. However, these schemes cannot further track moving vehicles with more accuracy and computational efficiency. Thus, Xiao et al. [6] presented a fusion approach of using radar and monocular cameras for robust detection and tracking. However, it needs additional computational and cost overheads to adopt the camera system together.

Despite the above advantages of previous works, when the radar sensor is deployed in tunnel environments, it also shows significant performance deterioration due to unexpected signal reflection and diffusion caused by the surrounding walls and the ceiling of the tunnel. This performance reduction in the radar sensor causes misrecognition of vehicles and surrounding objects, which significantly increases the risk of accidents if the driver or the control personnel use the wrong information. Although these performance degradation phenomena and models have already been explored in References [8,9,10,11,12], the problem is not yet fully resolved. Recently, a few studies on various clutter structures in road environments [13,14,15,16] have been conducted to improve radar performance for vehicle detection. In Reference [13], the authors suggest a new method for tracking stationary objects (e.g., guard rails and concrete walls) in front of a vehicle, making use of echoes from a typical vehicular radar. In Reference [14], the authors present a bridge identification algorithm based on the interference pattern resulting from the multipath propagation of the radar signals. The authors of Reference [15] present a technique for measuring the distance between two trains following one another through a tunnel at a distance of several kilometers. The authors of Reference [16] present a mapping technique based on measurements from automotive radar to enhance automotive safety functions, for instance trajectory control. However, these works mainly focus on the identification of bridges, guardrails or railway structures and do not consider vehicle tracking in the tunnel. Moreover, they still do not overcome tunnel clutters, which significantly reflect most kinds of wireless signals. Meanwhile, Lee et al. [17] proposed an iron tunnel recognition scheme to detect vehicles, especially in iron tunnel clutters by adopting the entropy concept and a spectrum spreading scheme. It analyzes the spectrum characteristics and intensive scattering over a wide frequency band. However, it is difficult to apply to concrete tunnels because it mainly deals with metal based tunnels. In addition, the target method is designed for the use of automotive purposes such as Adaptive Cruise Control (ACC) and Advanced Emergency Brakes (AEB), which are not directly incorporated into the roadside radar system.

There are also many related works that explore a ghost target problem [12] by using enhanced signal processing schemes [18,19,20,21,22]. Rohling et al. [18] presented a waveform scheme with 3 different antenna beams to overcome ghost images and improve the measurement time. Miyahara et al. [19] proposed another algorithm for multiple target detection in Frequency-Modulated Continuous-Wave (FM-CW) radar. It provides the distance and relative velocity of vehicles without the ambiguity of distance and relative velocity, an inherent problem of FM-CW. In Reference [20], a combination of linear frequency modulation (LFM) and frequency shift keying (FSK) is proposed to provide a high range and velocity resolution, a short measurement time and to mitigate any ghost target problems. Bi Xin et al. [21] also proposed a waveform, combining LFMCW with SFCW pairing to improve the measurement time. Jau-Jr Lin et al. [22] adopted the three-segment waveform and proposed a baseband signal processing architecture to satisfy the short measurement time constraint without increasing the RF front-end loading. Although these methods can obtain both the accuracy and the short measurement time in order to tackle the ghost image problem, they mainly focus on RF front-end design and do not deal with the noise filtering over the application layer. In addition, they also do not guarantee good detection performance in tunnel environments. To the best of our knowledge, this paper is the first attempt to deal with noise filtering in tunnels at the application level of the radar system.

In this paper, we propose an optimal installation method that can analyze the movement of the vehicle by installing a radar on the roadside. The proposed method derived the best installation parameters with the highest recognition rate of vehicles by tuning the height, tilt angle, and direction and considering the general features of a commercialized radar sensor (e.g., Delphi ESR). In addition, we analyze the factors of the performance degradation phenomenon of the radar signal in the tunnel space and then propose a signal processing and noise filtering algorithm to overcome it. The proposed noise filtering algorithm minimizes recognition failures based on vehicle speed, radar update rate, system queue, internal threshold value and so forth. In order to verify the performance, the radar was installed in a real vehicular tunnel to confirm the detection accuracy of moving vehicles.

The organization of this paper is as follows: In Section 2, we present the installation plan and experimental results for the optimal performance of the radar system with the roadside installation; Section 3 presents the detailed signal filtering algorithm and the experimental results for the operation of the radar in the tunnel; Finally, the concluding remarks and future works are presented in Section 4.

## 2. Radar Application for Roadside Deployment

### 2.1. Design of Road Watch Module

**Figure 1:**
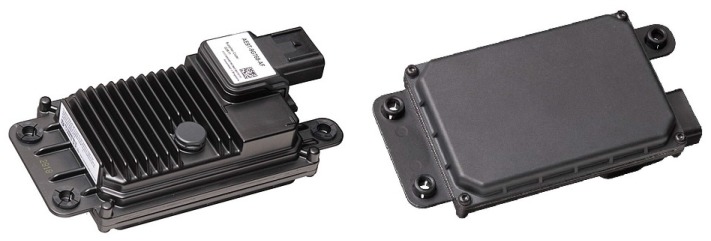
Delphi Electronically Scanning Radar (ESR) Platform.

For accurate and stable vehicle traffic analysis on the roadside, the radar sensor is commonly used as the main detection equipment around the world. The type of radar varies according to the frequency band and the modulation method. Among the various radar sensors, however, we design an efficient deployment method for optimal detection performance on the road by using Delphi’s ESR radar [23], which is currently one of most popular products for vehicle detection and has excellent price competitiveness. The radar platform and detailed specifications are shown in Figure 1 and Table 1, respectively.

In this study, a road watch module (RWM) based on ESR was designed. The main purpose of this module is to detect stationary and moving vehicles within the detection range of the radar sensor provided by the radio frequency (RF) signal of an antenna device. It also detects the position, lane, and speed information of the target vehicle. The vehicle to be monitored and the target road environment are selected as follows. The width of the vehicle is selected to be 1–2.5 m in reference to the sedan and the recreational vehicle (RV), which are the most popular vehicles for general people. The number of lanes in the road is 2–4 with a width of 2.5–3 m. The radar platform is installed on the roadside and the installation height is set to 2.5–3 m, which is the average height of the roadside traffic sign. Meanwhile, this installation height is different with the case of automotive radar due to the fact that the installation height of the radar in the vehicle is 60 to 100 cm, which is the height of the vehicle bumper.

**Figure 2:**
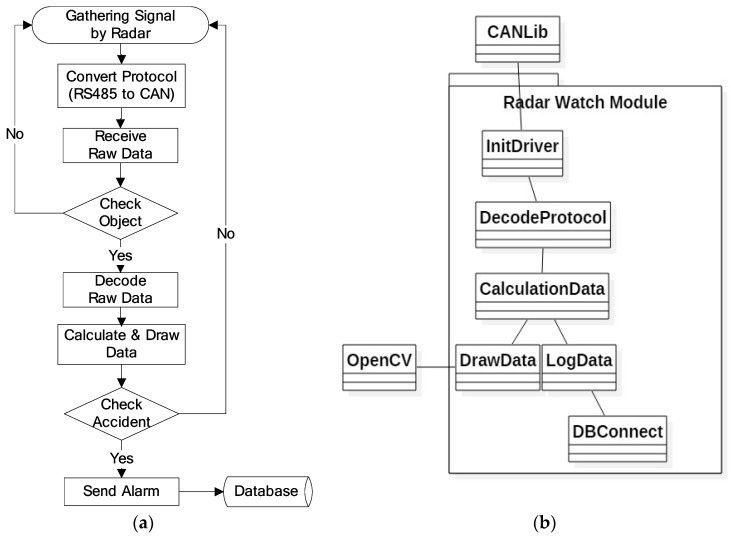
Overall architecture of the Road Watch Module (RWM): (**a**) Operation procedure; (**b**) module diagram.

After the installation of the radar, the user application connected to the radar should interpret the detection data streams obtained from the RF, and the RWM performs the function of acquiring and decoding these detection data streams. For this, the RWM and the radar sensor communicate with each other based on the Controller Area Network (CAN) protocol [6], which transmits bit stream through the network interface. After decoding the data stream, RWM can distinguish stationary objects (e.g., buildings, trees, road signs) from moving vehicles by adopting a real-time speed comparison algorithm. In addition, it also maintains a text log component that stores a raw data stream in real-time, so that it is possible to confirm that the RWM successfully receives every data stream provided at an update rate of 50 ms. The detailed operation sequence and module diagram are shown in Figure 2a,b respectively.

**Figure 3:**
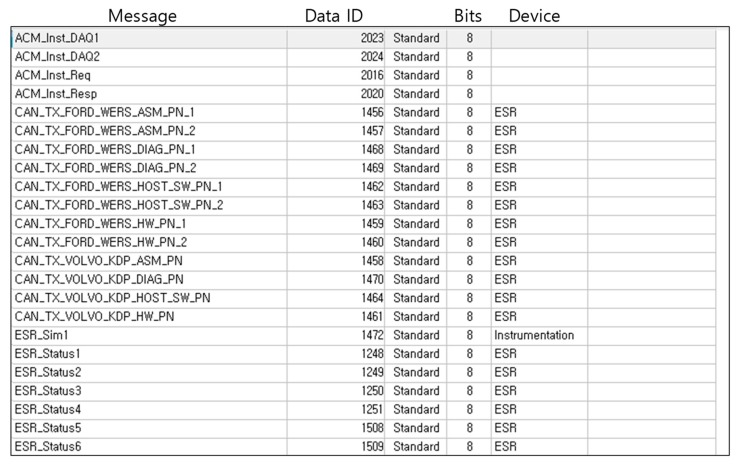
Example of excluded information in the Object Identification component.

The overall operation flow of the RWM is a series of functional iterations which are performed in the following order: radar signal collection, protocol conversion, raw data reception, object identification (ID grant), raw data decryption, data calculation, accident check (e.g., abnormal vehicle detection), abnormal signal notification and database storage. In the radar information collection function, RWM collects a data stream including the distance, speed, and length of the object detected by the radar. In protocol conversion, the RWM converts the incoming radar data provided by the RS485 communication protocol into the controller area network (CAN) protocol. The “raw data reception” component receives and inserts the raw data, which is then delivered by the CAN interface into the RWM buffer. In the “object identification” component, the RWM assigns a unique ID to an object identified as a detected target among the received raw data. That is, the object to which the ID is assigned becomes a moving vehicle or an obstacle (clutter) to be monitored. If the data is not judged to be a target object (e.g., radar management data) to be monitored, it moves to the radar signal collection section and then it repeats the signal collection process. The example information that is excluded from the set of monitoring objects is shown in Figure 3, where the representative one is digital to analog (D/A) converted information, data acquisition (DAQ) related information, device diagnosis related information and so forth. For more detailed information on radar data, please refer to the Kvaser database [24]. 

**Figure 4:**
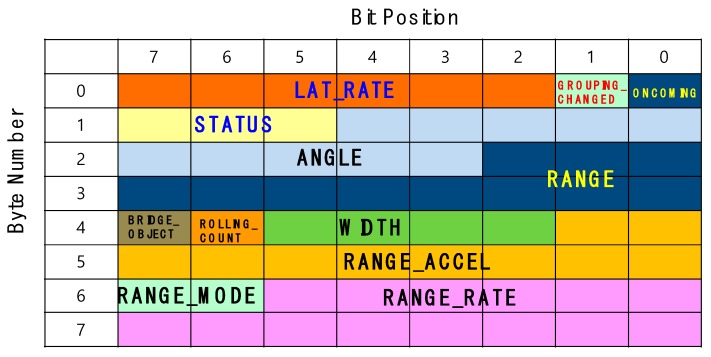
Bit map for radar data.

The main advantages of the proposed RWM are summarized as follows:

(1) Generally, the objects’ information that is obtained from the radar is very diverse and the amount of information is also very large. This large amount of data is still a burden to process in real-time on embedded devices connected to the radar. For this purpose, RWM preferentially inputs only those attributes that are relevant to the moving vehicles, minimizing the response latency by filtering meaningless information. In particular, since Delphi ESR radar only provides bitmap information of raw data, as shown in Figure 4, and does not provide data selection, acquisition, or communication methods for specific applications, the RWM provides a framework for the analysis and processing of the raw data from the radar.
(2) With respect to the vehicle information obtained, only the vehicle located in the road is tracked with reference to the width of the road, the number of lanes and the length of the lane, so that the processing delay time can be reduced and detection accuracy can be enhanced.
(3) In addition to accurate vehicle identification on the road, the RWM also provides a noise filtering technique in tunnels by analyzing various false signal patterns such as ghost phenomena and flickering. The proposed scheme provides robust detection accuracy and low calculation latency in tunnels.
(4) By adopting the RWM technique, it is possible to confirm the occurrence of an accident by analyzing the stop pattern and congestion phenomenon of a vehicle.

In the “raw data decoding” component, the RWM application extracts necessary information by decrypting the raw data stream. That is, the meaningless raw data acquired from the radar is converted into high level information which is utilized at the application level. A bit-map of the data protocol used for decoding is shown in Figure 4. 

The above bitmap is composed of 8 bytes and the information about the target vehicle’s motion such as the speed, direction, and distance is as follows:

− ANGLE: Angle of the RF signal that is reflected from the target object after the radar transmits it.
− LAT_RATE: Speed of an object moving in the horizontal direction between lanes.
− RANGE: Distance between the radar and the target object.
− RANGE_RATE: Relative speed between the radar and target object.
− RANGE_WIDTH: Width of the target object.

**Figure 5:**
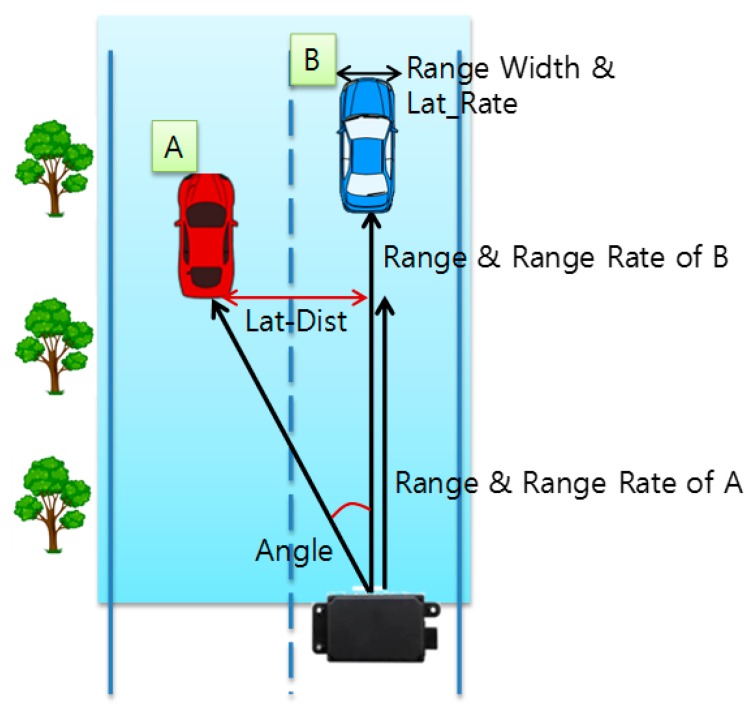
Extracted information from vehicular radar.

Figure 5 shows an example of the measurement of the moving vehicle on the road using the extracted information via the RWM. In Figure 4, if the radar is installed in the middle of the road or the radar is mounted on the front bumper of the vehicle, the vertical distance between the preceding vehicles A and B, the relative speed between radar and vehicles and the angle with respect to the forwarding direction can be known. However, since the ESR radar is mainly concerned with the presence or absence of the front obstacle according to adaptive cruise control (ACC) or advanced emergency brake (AEB) operations, the horizontal distance between lanes for vehicle A is not explicitly provided. Nevertheless, because ANGLE values can be checked, we can easily find Lat-Dist by using the following equation:

(1) $$\mathrm{Lat}-\mathrm{Dist}=\mathrm{Range}\times \mathrm{Tan}\;(\mathrm{ANGLE}\times \frac{\pi }{180})$$

**Figure 6:**
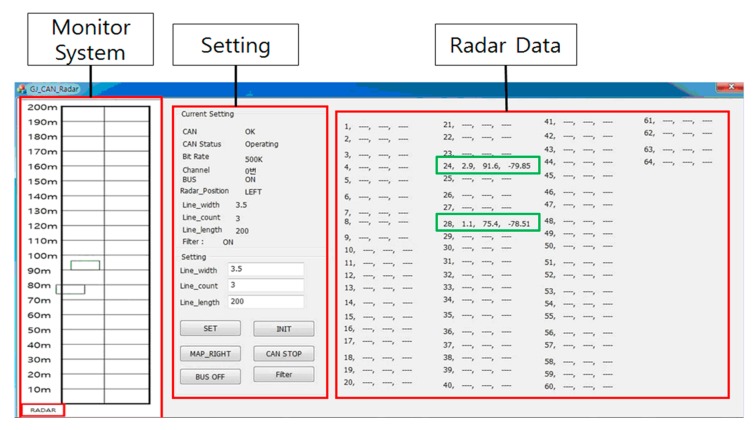
Graphical User Interface (GUI) for range configuration and display.

The “calculate & draw” component has the role of verifying whether the detected object is located in the current lane or road, based on the coordinate information obtained through Equation (1) and the information on the physical environment of the road. After finishing the verification of the target object, it also provides a function of displaying corresponding object information in the graphical user interface (GUI). For this, the user can first input the information of the target road on which the radar is installed. As shown in Figure 5, three pieces of environmental information (such as the width of the road, length of road and the number of lanes) to be monitored are set through the main GUI provided by the RWM. However, the length of road, which is measured and displayed, does not exceed the maximum monitoring distance (Range_Max) of the radar. In the case of Delphi ESR radar, Range_Max is defined as 174 m. Through the three pieces of input information, we can set the target zone to be monitored by the radar, and it is easily calculated with the following equation,

Zone (X_n_, Y_n_, X_n+1_, Y_n+2_) = (0, 0, LW × LC, LL), LL ≤ Range_Max (2)

where, LW, LC, and LL denote the lane width, lane count and lane length of the target road, respectively. The calculated target zone is within the range of the blue rectangle in Figure 4. In addition, by tracking only moving objects within this range, it is possible to improve the calculation speed and increase the recognition rate of the vehicle by excluding the reception of unnecessary clutter information such as trees, buildings, street lamps and so forth. In Figure 4, the objects recognized as vehicles in the range of the radar are displayed in square form through the OpenCV library [25] on the target zone. Vehicle A and Vehicle B in Figure 4 are assigned ID 28 and ID 24 in Figure 5, respectively, and they are shown in green squares. In addition, the Lat-Distance, Range, and Velocity for each vehicle are displayed in the ‘Radar Data’ field on the right side of the GUI for specific object movement information. The velocity is marked as ‘+’ when moving away from the installed Radar, and the approaching object is marked with ‘−’. In Figure 5, the two vehicles—ID 24 and ID 28—are approaching at 79.85 km/h and 78.51 km/h, respectively. In the case of ID 24 in Figure 5, the data set of [24, 2.9, 91.6, −79.85] denotes that the vehicle’s ID is 24, Lat-Distance is 2.9, Range is 91.6 and velocity is −79.85. At this time, it is important to note that the three trees shown in Figure 5 are excluded from Figure 6 because they are outside the target zone. Meanwhile, a complicated section where the radar cannot overcome a non-line of sight (NLOS) condition, such as a curved road or an intersection, can be monitored by overlapping a plurality of radars.

The “check accident” component provides a function that warns the user of a vehicle accident. Although there are many kinds of vehicle accidents, the main accident is defined as a case in which the vehicle is unexpectedly stopped on the road due to a collision or breakdown. To analyze the accident stop pattern, the RWM uses the following conditional equation,

**Condition** **1.** $P_{cur(t)}^{i}\in$ *Target_Zone*

**Condition** **2.** $V_{avg(t,t+\theta )}^{i}$ = 0, *θ* > 0

$P_{cur(t)}^{i}$ is the position of vehicle *i* at current time *t*. Condition 1 determines whether the vehicle is within the target zone of the radar. $V_{avg(t,t+\theta )}^{i}$ represents the average speed of vehicle *i* during the period from the current time *t* to *t* + *θ*. Condition 2 confirms whether the vehicle is in a stopped state (i.e., velocity is 0 km/h) during the threshold time (*θ*). In this study, *θ* is set to 30 s although it depends on traffic conditions and traffic regulations on the target road. Of course, it can be set shorter on the road for the exclusive use of vehicles (e.g., expressway). If the vehicle is stopped for a certain period of time using the above two conditions, an accident may be suspected. However, if vehicles are backed up due to a road bottleneck phenomenon, the vehicle will inevitably be stopped. In this case, it is difficult to distinguish an accident from a bottleneck. To resolve this problem, we use Conditions 3–5 below. 

If there is not a large-scale collision accident, it is regarded as a bottleneck. Since the bottleneck phenomenon means that all vehicles are stopped, this condition is expressed as Condition 3, as follows.

**Condition** **3.** $\sum_{i=0}^{i}V_{cur(t)}^{i}=0$

Generally, after a certain period of time, the preceding vehicles continue to move as the bottleneck is alleviated. At the same time, the *i*-th vehicle also starts to move at a higher speed to deviate from the area of interest. In this case, it is recognized as a normal traffic pattern or a simple bottleneck rather than an accident. On the other hand, although the preceding vehicle has started moving and has departed from the target zone, vehicle *i* is judged to be an accident vehicle if the vehicle *i* is kept in the stopped state for a critical time. In this situation, the movement of the preceding vehicle except for vehicle *i* is expressed by Condition 4.

**Condition** **4.** $\sum_{n=0}^{i-1}V_{avg(t,t+x)}^{n}>0$

where *x* is the time until the bottleneck disappears. The time can be measured in real-time via the vehicle’s flow in the radar or by manual input. The condition where vehicle *i* is stopped for the critical period after the bottleneck release point is expressed in Condition 5 as follows.

**Condition** **5.** $V_{avg(t+x,t+x+\theta )}^{i}=0$

As a result, if the above five conditions are satisfied, the RWM confirms that a bottleneck phenomenon is not currently present, and transmits the coordinate information (Range, Lat-Distance) of vehicle *i* to the user along with an accident judgment message. After an accident alarm, the corresponding information is recorded in the database in the “DB storage” component.

### 2.2. Derivation of Optimal Performance for Roadside Deployment

In order to evaluate the performance of the developed RWM and to derive the optimum performance conditions, the vehicle detection accuracy was measured. The detection accuracy was calculated as the average of two accuracy results, which include the accuracy of a moving vehicle (Accuracy_MV) and the accuracy of a stopped vehicle (Accuracy_SV), assuming an accident. These measurement environments are expressed by Equations (3)–(5) as follows:

(3) $$\mathrm{Vehicle}\;\mathrm{detection}\;\mathrm{accuracy}=\frac{\mathrm{Accuracy}\_\mathrm{MV}+\mathrm{Accuracy}\_\mathrm{SV}}{2}$$

(4) $$\mathrm{Accuracy}\_\mathrm{MV}=\frac{\mathrm{Correctly}\;\mathrm{detected}\;\mathrm{number}\;\mathrm{of}\;\mathrm{moving}\;\mathrm{vehicles}}{\mathrm{Total}\;\mathrm{number}\;\mathrm{of}\;\mathrm{moving}\;\mathrm{vehicles}}\times 100\%$$

(5) $$\mathrm{Accuracy}\_\mathrm{SV}=\frac{\mathrm{Correctly}\;\mathrm{detected}\;\mathrm{number}\;\mathrm{of}\;\mathrm{stopped}\;\mathrm{vehicles}}{\mathrm{Total}\;\mathrm{number}\;\mathrm{of}\;\mathrm{stopped}\;\mathrm{vehicle}}\times 100\%$$

**Figure 7:**
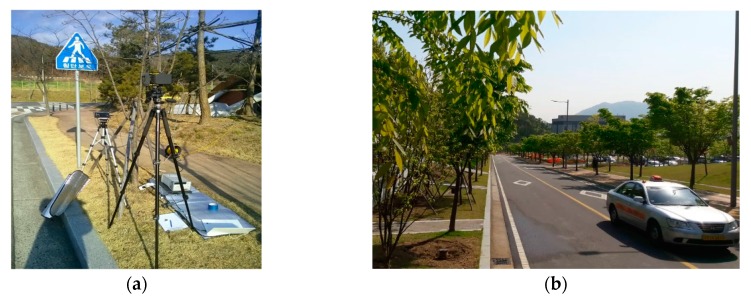
Radar installation for roadside surveillance: (**a**) Radar with stand holder; (**b**) target road and vehicle.

**Figure 8:**
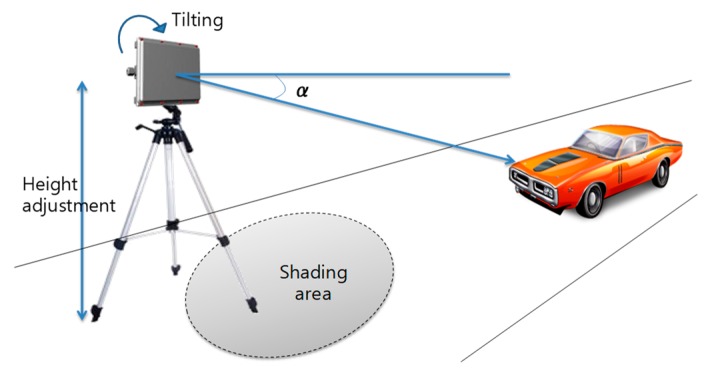
Radar configuration with angle (α) and height.

The target vehicles for the experiment were randomly selected on the campus road. For vehicle accident simulation, one sedan type vehicle was selected. To measure the detection accuracy, the selected vehicle was repeatedly run and stopped more than 50 times and the number of correct detections was measured. In order to increase the detection accuracy for the vehicles by installing the radar on the road surface, it is necessary to tilt it slightly in the forward direction of the road in consideration of the straightness of the RF signal of the radar. It is also necessary to set the high positioning of the radar for extending the detection range. Based on these facts, the vehicle radar is mounted on a dedicated cradle and placed on the roadside, as shown in Figure 7. We then measured the detection accuracy while manually adjusting the height of the mount, the installation angle, and the number of target vehicles, as shown in Figure 8.

**Figure 9:**
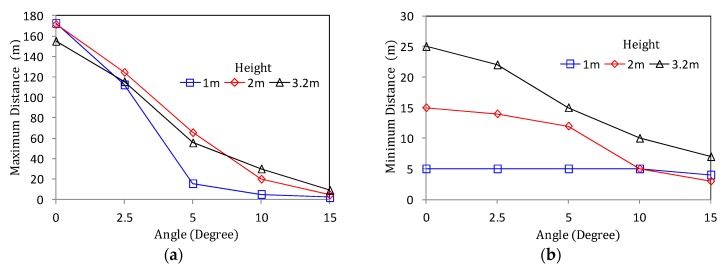
Detection distance of RWM according to radar angle: (**a**) Maximum detection distance; (**b**) minimum detection distance.

Prior to measuring the detection accuracy, we measure the maximum distance at which the vehicle is correctly detected according to the tilting angle α and the height of the radar installation, which is illustrated in Figure 9a. In general, as the tilted angle becomes larger, the RF is directed towards the road surface, which means that the maximum distance to detect the vehicle becomes shorter. Especially, in the case of 1m height, the detection distance sharply falls when the angle is over 2.5°. Moreover, when the angle is greater than 5°, the detection distance is less than 20 m, which makes it difficult to monitor the traffic on the road. This means that the vertical field of view (FOV), in which an object can be recognized, sharply decreases.

On the other hand, Figure 9b shows the minimum detectable distance based on the radar. It can be seen from the figure that the higher the installation height, the more signal shaded areas are generated below the radar where vehicle detection is impossible. In the case of 3.2 m and 2 m height, if the angle is 0°, the front shadow length of the radar is 25 m and 15 m, respectively. To overcome this long shadow area, it can be reduced by increasing the tilt angle. Whereas, in the case of 1 m height, the variation of the minimum detection distance is less than that of the angle adjustment. If only the detection distance comparisons of Figure 9a,b are taken into consideration, then a low installation height and a low tilted angle can be accepted as reasonable installation conditions.

**Figure 10:**
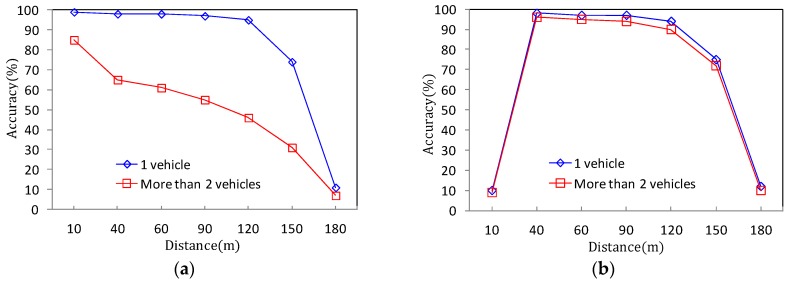
Detection accuracy of RWM according to vehicle distance (α = 2.5): (**a**) 1 m height of radar installation; (**b**) 2 m height of radar installation.

However, it is important to note that simply setting α lower than 2.5° does not always guarantee its performance. Figure 10a shows the detection accuracy of the RWM according to the number and distance of the monitored vehicles on the road at an installation height of 1 m. In the case of one target vehicle, the detection accuracy is more than 95%. However, when two or more vehicles are overlapped within the detection range of the radar, the performance sharply drops, and the average accuracy is approximately 60%. Since the height of 1 m is less than the actual height of the vehicle (i.e., averagely 1.5 m above the ground), the preceding vehicle is not detected by the radar due to the NLOS problem. That is, the preceding vehicle is hidden by the rear vehicle. As a result, the height of 1 m or less only can be adopted to the application of the ADAS system such as front obstacle detection or collision risk warning, but the performance is insufficient for road traffic monitoring on the roadside.

Meanwhile, in Figure 10b, the same experiment is performed at a height of 2 m. The result reveals that even when two or more vehicles are moving, the recognition performance is not significantly decreased below that in the case where only one vehicle is moving. In addition, according to Figure 9a, when the height is 2 m or 3.2 m, it is easy to reserve the wide vertical FOV. Therefore, the reduction of the detection distance performance by the tilting angle is relatively small compared to using a height of 1 m. However, because of the shadow area of around 15 m which is observed in Figure 9b, the detection accuracy is also significantly decreased (less than 10%) within a 15 m distance. Of course, it can be seen that the performance of detection accuracy suddenly drops even when the maximum detection distance of 120 m derived from Figure 9a is exceeded.

As a result, the experimental results of Figure 9 and Figure 10 show that the best performance, that is, the highest detection accuracy and the longest detection distance can be obtained when the radar is installed on the roadside with the tilted angle of around 2.5° at a mounting height of around 2 m.

## 3. Radar Application for Tunnel Deployment 

**Figure 11:**
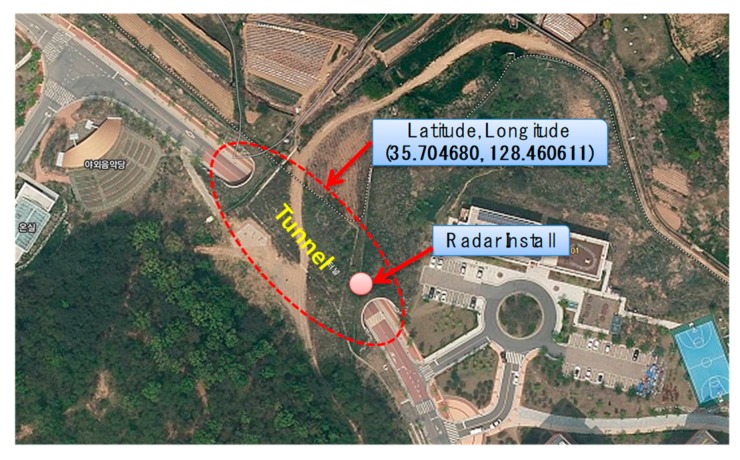
Target tunnel.

**Figure 12:**
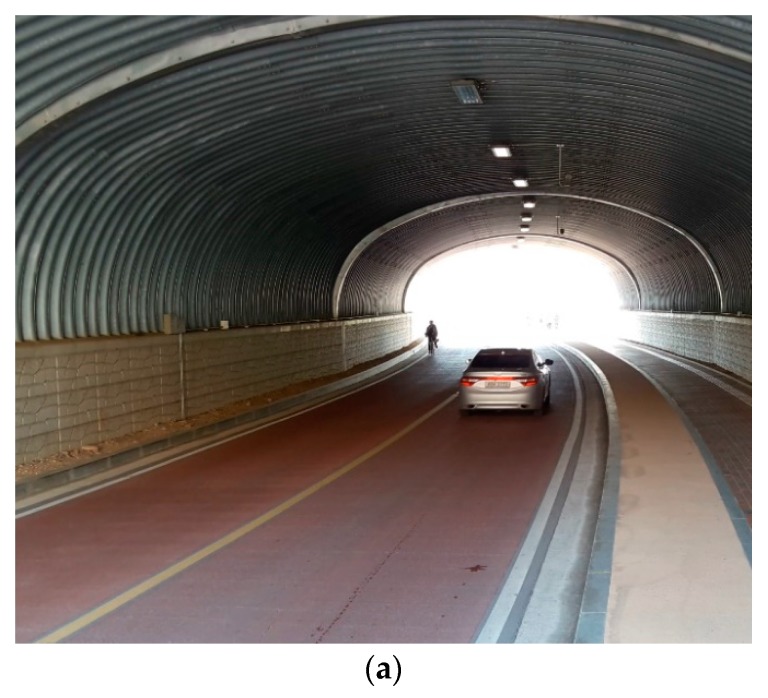

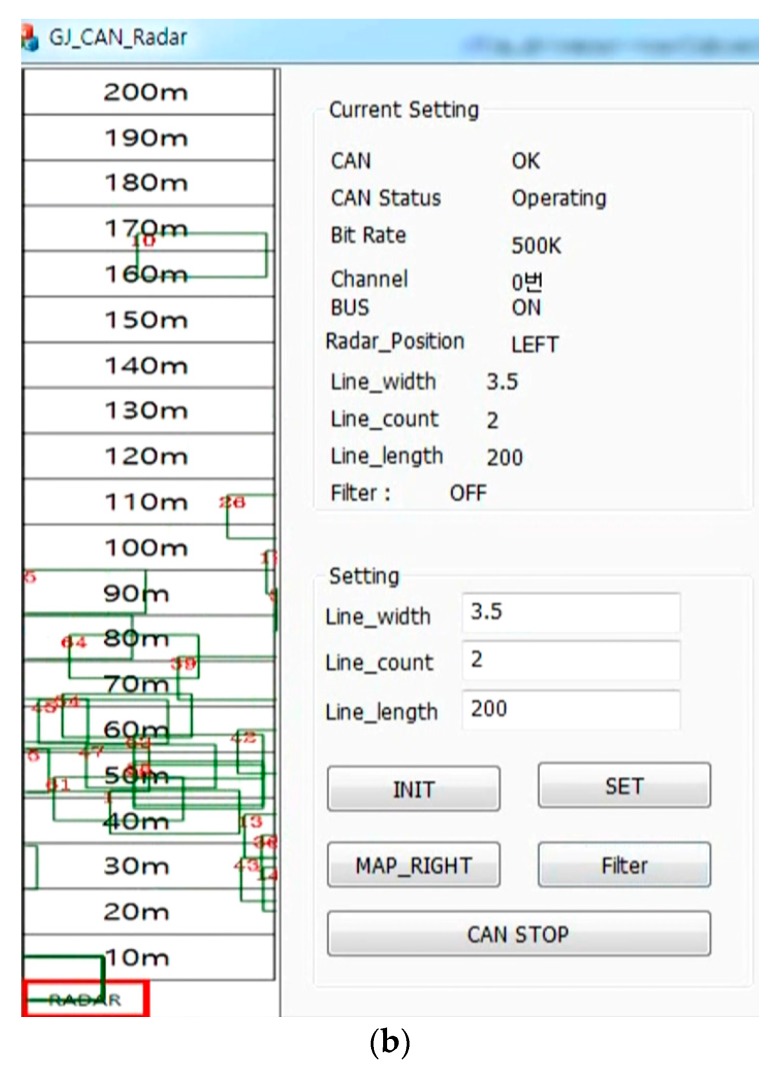
Radar installation in tunnel: (**a**) Vehicle in tunnel; (**b**) measured data.

After the optimal installation height and installation angle for the road monitoring were found using the empirical method in Section 2, this section confirms experimentally whether it is possible to monitor the vehicle in the tunnel environment where the signal reception ratio and detection accuracy generally show poor performance. As shown in Figure 11, the target tunnel was selected from the DGIST campus located in Daegu city, Republic of Korea, and the radar was installed near the entrance of the tunnel. The length of the tunnel is 80 m, the width is 13.5 m, and the height is 6.5 m from the ground. The experiment verifies that the position and velocity data are accurately measured during the movement of the vehicle at a speed of 30–60 km/h from the entrance to the exit of the tunnel. As shown in Figure 12, unfortunately, the experimental results show that even though a single vehicle passes through it, the radar system detects incorrectly by dozens of vehicles. This is because the radar RF signal causes diffuse reflection by the enclosed structure such as the walls and the ceiling of the tunnel. In this situation, the transmitted signal is misunderstood as a plurality of vehicles by receiving not only the reflected signal from the vehicle but also the reflected signal from the tunnel structure. In order to identify the noise pattern in the real tunnel, we drove a sedan type vehicle, shown in Figure 12a, and repeatedly entered the tunnel about 100 times. As a result of repeated experiments, the main features of the diffuse signal (noise patterns) are summarized as follows.

(1) It is recognized as if the vehicle is running at an incorrect location beyond the width of tunnel and the road. It is also called a ghost image.
(2) It is recognized as if the vehicle in the tunnel instantly achieves a high speed status that is out of the realm of common sense (e.g., 290 km/h or faster) or achieves a rapid acceleration status.
(3) Even though the actual vehicle runs along the road, the distance between the radar and the vehicle does not increase. It is recognized as being the same or decreasing (recognition as stopping or backward/reverse run).
(4) The vehicle suddenly appears and disappears within one second, and this phenomenon is repeated several times or continuously as if it is a flicker.

**Figure 13:**
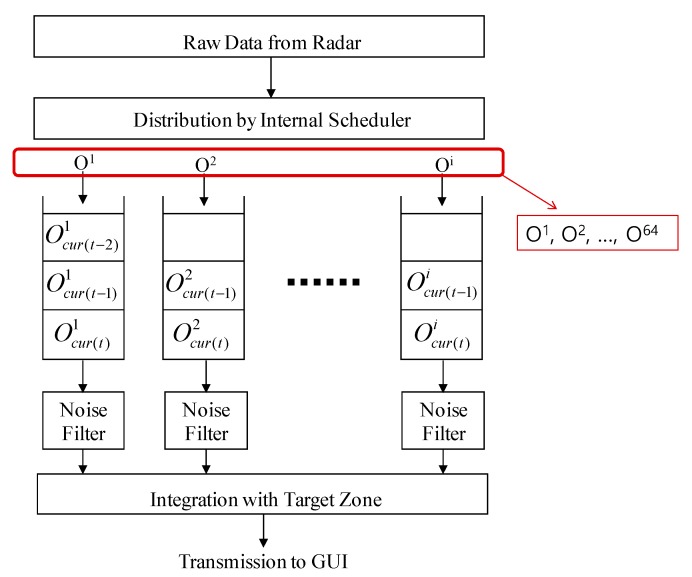
Separate queue architecture of the RWM.

In order to resolve the aforementioned problems and robustly monitor the moving vehicles in the tunnel, the RWM maintains an object tracking table (OTT) which consists of the following set: {Object ID, Range, Range_Rate, Lat-Dist, Lat_Rate, Vehicle_Possibility, Is_Vehicle and *T*_EX_}. The Object ID is the unique identification generated by the radar and assigned to every object including vehicle candidate and clutter. The RWM basically creates a total of 64 objects, $O^{i}$ (1 ≤ *i* ≤ 64), as shown in Figure 13 by referring to general commercial radar products (e.g., Delphi ESR). Individual objects that are recognized as running vehicles are stored in independent queues, which enables the RWM to perform robust detection such as individual location tracking, driving pattern analysis and noise filtering. In the general radar system, the corresponding speed and location information of each object is updated in 50 ms units, and the previous data is automatically dropped from the queue. However, for noise filtering, each queue of the RWM separately maintains two additional pieces of information—$O_{cur(t)}^{i}$ and $O_{cur(t-1)}^{i}$—which represent the object data of $O^{i}$ at the current time *t*, and the object data before the 50 ms unit time, respectively. Through these two pieces of information, it is possible to identify the location before and after each vehicle, and it is also helpful to confirm that the vehicle is in the correct lane. However, since the capacity of the target system’s memory and the calculation performance are different from each other, the number of objects to be maintained per unit of time may vary. In the case of the RWM, at least two pieces of object information are needed to be maintained for the state before and after each unit of time (e.g., 50 ms). Meanwhile, the definitions of Range, Range_Rate, Lat-Dist and Rat_Rate in the OTT are already described in Figure 3 and Figure 4 of the previous section. Vehicle_Possibility denotes a judgment weight value for distinguishing whether the vehicle is actually running on the road to mitigate the noise. Is_Vehicle is a flag value, which records the judgment result of whether the vehicle is an actual vehicle. *T*_EX_ is a timer that recognizes an object as a vehicle and keeps this information in an OTT for a specified period of time. It means that after a certain period of time specified in *T*_EX_, the information can be deleted from the memory for resource management. Of course, if the vehicle leaves the target zone or is recognized as clutter, it is deleted from the OTT.

The solution algorithm of the RWM, using the OTT to tackle the four noise patterns mentioned above, is shown in Figure 13. Basically, the proposed algorithm judges whether each detected object is normal and the actual moving vehicle in the tunnel through a filtering procedure at every interval of 50 ms. If the object is determined to be a normal vehicle, the location of the vehicle on the radar map drawn on the UI is shown. If the object is determined to be a normal vehicle, the location of the vehicle is displayed on the radar map of the user interface. On the other hand, if the object is not confirmed as a vehicle, it is considered to be noise data caused by the influence of signal diffusion or a disturbance, and then the RWM immediately drops it from the memory. The detailed operation procedure is as follows. First, the above-mentioned noise signal pattern (1) can be easily resolved by setting the target zone as shown in Figure 4 and recognizing the vehicle outside the zone as noise. The noise pattern (2) is simply treated as noise and is dropped when the object moving faster than 290 km/h. Of course, it can be modified dynamically depending on the road environments and the driving characteristics of the vehicle. However, in this study, we assume that the speed limit of a general urban road is 60–80 km/h. For noise pattern (3), the speed of the object at current time *T* and the speed at the time of *T*-1 both exceed 0, and when the travel distance is longer than 0, it is recognized as a normal driving vehicle. Then, all other abnormal driving patterns are regarded as noise and are dropped. In order to solve the last noise pattern (4), the Vehicle_possibility value is used. It determines the possibility of whether or not it is an actual vehicle based on an accumulated pattern recognition. Its initial value starts from 0 and increases by 1 when all the noise patterns (1) to (3) are satisfied. Thereafter, when Vehicle_possibility exceeds a certain threshold value *β*, it is recognized as an actual vehicle at the moment and the result is displayed on the UI. The threshold value, *β* is defined as the number of times the radar information is updated to recognize the object as one vehicle.

For example, suppose a typical 5 m long vehicle runs at 8.33 m/s (30 km/h). The first recognition of the radar is performed by reading the position value of the reflection point of the front bumper or the rear bumper of the vehicle and judging it as an object. Therefore, it takes about 0.6 s to measure the moving time of the vehicle by 5 m after the first recognition. Since the data update rate of the radar is 50 ms (0.05 s), it recognizes the object about 12 times in 0.6 s. Therefore, if the RWM sets the threshold value to 12 and consecutively recognizes 12 times as the same object, it is judged to be a normal vehicle. If the vehicle travels at a speed of 80 km/h (22.2 m/s), the travel time by the vehicle average length is about 0.22 s. During this time, the radar will recognize it 4.5 times, thus the threshold is set to 5. This can be summarized as follows.

(6) β=Vehicle_width(m)Vehicle_velocity(m/s)Update_rate(s)

**Figure 14:**
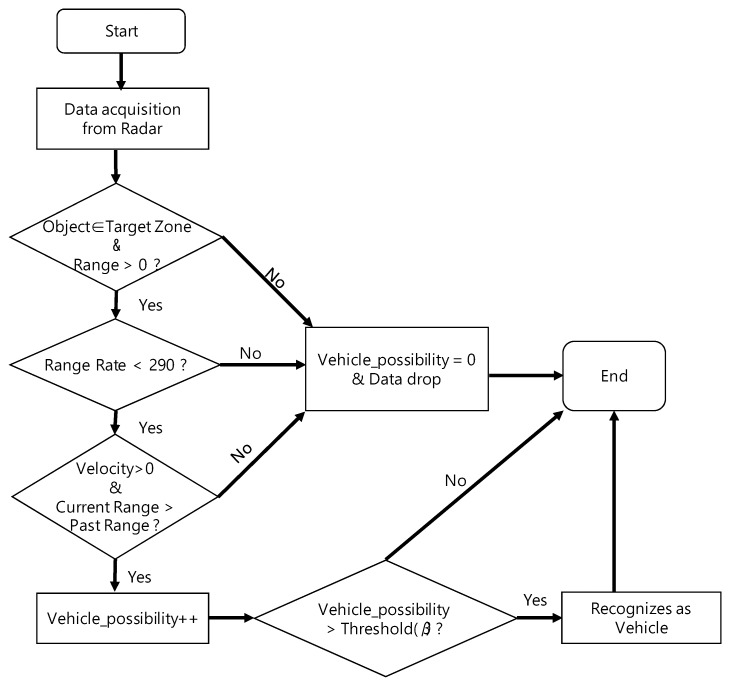
Noise filtering algorithm of the RWM.

**Figure 15:**
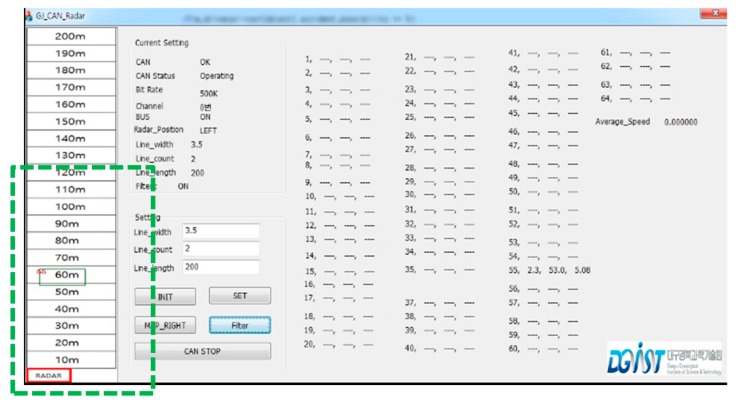
Target detection with noise filtering.

An overall flow chart of the noise filtering algorithm is shown in Figure 14. In addition, Figure 15 shows that the vehicle is correctly recognized when the filtering algorithm is applied to the result of the vehicle misidentification in the tunnel shown in Figure 11.

**Figure 16:**
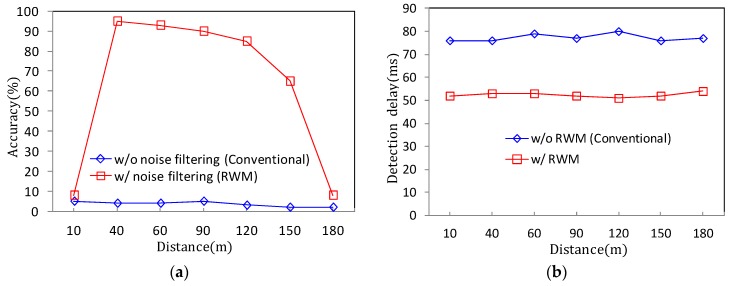
Vehicle detection accuracy and delay performance comparison: (**a**) Accuracy performance according to target distance; (**b**) delay performance according to target distance.

For a practical performance validation, an experiment was conducted on randomly selected vehicles moving at speeds of 30 to 60 km/h in the tunnel shown in Figure 11 and Figure 12. The installation height of the radar was set to 2 m from the ground using the same method as presented in Section 2, and the tilt angle was maintained at 2.5° in the ground direction. In the experiment, the detection accuracy of the vehicle in the tunnel and the detection delay time are compared. The results are summarized in Figure 16. Figure 16a compares the case where noise filtering is applied according to the distance that the target vehicle moves away from the radar. Figure 16b measures and compares the delay time when the target is updated and recognized in the RWM. As shown in Figure 16a, the proposed noise filtering technique has higher accuracy than the conventional technique without filtering. This is because the existing technique fails to overcome the problem of diffuse reflection in the tunnel, which results in many false positive errors. For example, one vehicle is mistaken for several vehicles, or even if the vehicle has already passed through the tunnel, the radar cannot distinguish between the tunnel wall and the absence of the vehicle. On the other hand, the proposed scheme achieves higher than 80% recognition accuracy up to a 120 m distance by minimizing errors through filtering of the signal and achieves more than 90% performance in the 40–100 m distance range. Although the data obtained in this experimental study are obtained by observing more than 1000 moving vehicles, there are important issues to consider when conducting such an experiment on actual roads and in tunnels. Although the proposed noise filtering technique is efficient, the obtained object’s signal primarily depends on the radar RF. The RF signal is especially highly affected by the length, width, height, and material of the tunnel surface as well as the vibration due to wind during operation. In this experiment, the performance test is conducted by minimizing the external environmental factors and the influence thereof, but it may not be generalized equally to all environments. However, note that at least the proposed method can provide guidance how to detect and improve the accuracy at the user program level rather than the RF level.

**Figure 17:**
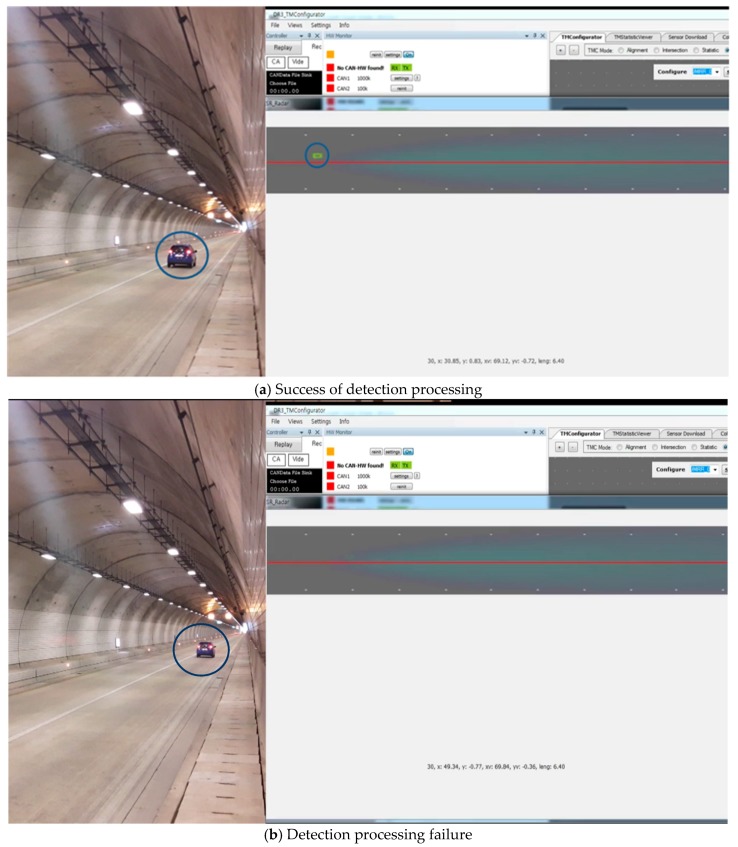
Example of detection processing delay.

In Figure 16b, the proposed filtering scheme shows a detection time of approximately 50 ms while the conventional technique shows a delay of 70–80 ms, which means that the proposed scheme is superior. Although the update time of incoming data from the radar interface is the same as 50 ms, there is a difference between the time to input and process the incoming data in the buffer of the sever computer and the time to display the final result in the application user interface (UI). The personal computer (PC) used in the experiment has 8 MB of memory. Note that although the proposed scheme adopts a separate queue control scheme for noise filtering, the conventional scheme outputs a considerable amount of noise, which causes a delay due to the data bottleneck. This can be a factor that hinders real-time recognition of vehicles. For example, a phenomenon occurs in which a certain vehicle disappears momentarily due to a real-time processing delay. This disappearing problem is described in Figure 17. Figure 17a shows that a vehicle is moving in the tunnel and is detected accurately through the radar UI, where the vehicle is marked with a blue circle. However, after a while, the vehicle disappears momentarily on the UI, even though the vehicle is still in the tunnel as shown in Figure 17b. However, the proposed RWM minimizes the processing delay by allowing a multi-task based queue structure to process multiple processes in parallel within a single computer.

**Figure 18:**
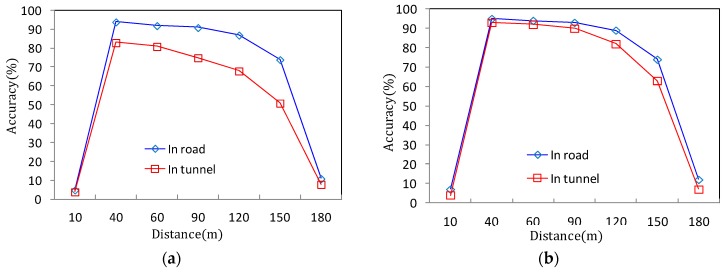
Accident recognition accuracy comparison: (**a**) Accuracy performance according to target distance (*θ* = 30 s); (**b**) Accuracy performance according to target distance (*θ* = 10 s).

We also conducted a performance evaluation of the RWM for accident recognition accuracy both on the road and in the tunnel, which is shown in Figure 18. Since we suppose that the accident recognition of the RWM depends on the time threshold value (*θ*), the experiments were carried out in two cases where *θ* is set to 30 s and 10 s. In both cases shown in Figure 18a,b, the accuracy performance on the road proved to be better than that in the tunnel. This is a pattern similar to that of the detection accuracy for moving vehicles. However, the overall performance for detecting an accident vehicle is lower than that of the moving vehicle. This is because the RWM misrecognizes that the vehicle has moved slightly due to the time difference in the instantaneous reception of the RF signal even though the vehicle is stopped. If the *θ* value for accident judgment is set as low as 10 s, Figure 18b reveals that the detection accuracy increases. This is a phenomenon in which the number of erroneously received signals is reduced by setting the judgment time to be short. As a result, to increase the accuracy of accident detection, it is necessary to shorten the judgment time or to further filter out the diffuse reflection signals that appear when the vehicle is stopped.

**Figure 19:**
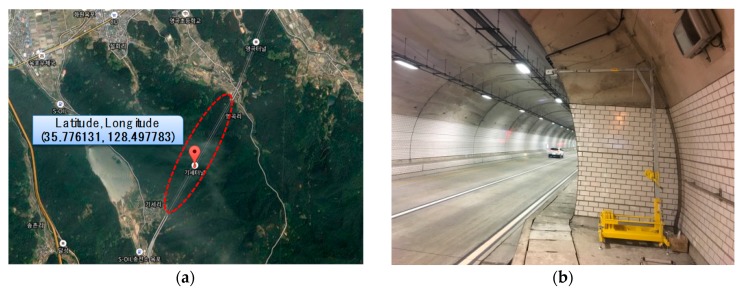
Experiments in urban highway: (**a**) urban highway (2.1 km) in Korea; (**b**) deployment of radar system in highway tunnel.

**Figure 20:**
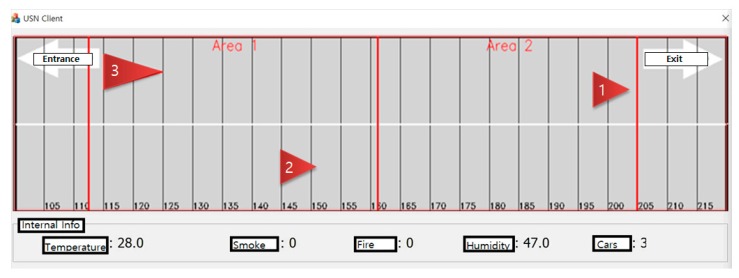
Traffic monitoring application using proposed radar system.

Meanwhile, for the deployment and management of large-scale tunnels rather than on-campus tunnels of 80 m size, the radar with the RWM was built in a commercial tunnel, as shown in Figure 18a. The target tunnel selected was the 1.9 km-long express tunnel (name: ‘Gise’ tunnel) located in Daegu city, Korea and the experiment took place under the same conditions as the previous experiment, using a special cradle as shown in Figure 19b (i.e., height: 2 m, tilt angle: 2.5°). However, the average speed of the vehicles was maintained at 70–80 km/h because it is a highway. The experimental results in the express tunnel showed good recognition accuracy and a recognition delay performance similar to the results in Figure 16 when noise filtering was applied. In addition, as shown in the Figure 20, it can be also used for monitoring vehicle traffic conditions at all times. In the figure, the red arrow indicates the recognized vehicle and the current direction of movement. Vehicle 1 and vehicle 3 are driving in the first lane, while vehicle 2 is driving in the second lane. In addition, the length of the vehicle can be checked using the radar, and it is identified that vehicle 3 is longer than vehicle 2 and vehicle 3.

There are a few consideration issues for running the RWM in tunnels. Since the proposed system tracks only 64 vehicles at the same time, it is difficult to track accurately in complex traffic situations where there are more than 64 vehicles moving simultaneously. In order to tackle this, it is necessary to adopt not only a radar device change but also a sophisticated software distributed processing algorithm. Finally, all target tunnels in this study are linear type tunnels where line of sight (LOS) is guaranteed. If a radar is installed in a curved tunnel, the detection performance for the vehicle will greatly deteriorate due to an increase in noise reflected by the facing wall.

## 4. Conclusions

In this paper, a radar data processing system named RWM was designed and implemented for vehicle traffic and accident detection in roads and tunnels. The RWM analyzes the general requirements of radar (e.g., Delphi ESR) and designs an architecture in consideration of road and tunnel environments. In particular, the optimal deployment conditions were derived by considering the installation height, tilt angle and so forth, which have the greatest influence on the performance of radar as environmental factors on the road surface. In addition, a noise filtering algorithm is proposed to overcome misdetection patterns due to diffuse reflection of the RF signal in the tunnel. And its performance was verified through various experiments with regards to detection accuracy and delay when it is compared to the conventional detection scheme of radar. By providing a user-friendly interface for the server application, it also can help traffic monitoring and accident response at the usual time.

For future work, we plan to analyze and compare the efficiency of various commercial platforms equipped with the RWM. We also expect that the proposed system will contribute to reducing traffic accidents in tunnels if the RWM and its operation program are installed in conventional vehicular radar units for roadside monitoring.

## Figures and Tables

**Table 1:** Specification of ESR Radar.

Parameter	Specification	Parameter	Specification
Frequency	76 GHz	Waveform	Pulse Doppler
Long Range	174 m	Mid-Range	60 m
Long Range Field of View (FOV)	+/−10 °	Mid-Range Field of View (FOV)	+/−45 °
Vertical FOV	4.2 °	Min. Amplitude	<−10 dB
Update Rate	50 ms	Max. Amplitude	>40 dB
Range Rate	−100–25 m/s	Range Rate Accuracy	<+/−0.12 m/s
Number of Targets	64	Min. Update Interval	20 Hz
